# Guidelines for the development of a dental emergencies educational workshop

**DOI:** 10.15694/mep.2017.000101

**Published:** 2017-06-14

**Authors:** Nozha Sawan

**Affiliations:** 1 Princess Nourah Bint Abdulrahma

**Keywords:** education, workshop, guideline, develop, design, dental, emergency

## Abstract

This article was migrated. The article was marked as recommended.

To draft any education workshop, several guidelines should be followed: 1.) describing the context; 2.) conducting a need assessment; 3.) developing the design and the delivery strategy; and 4.) evaluating learners and workshop. This paper presents the first general guidelines for drafting such an educational workshop that would target emergency medicine residents and physicians who are expected to acquire the knowledge and skills needed to properly manage various emergency situations of which dental emergencies is one of them.

## Introduction

Emergency residents cover various basic topics in medicine and surgery. In most health professional fields, including medicine, students undertake a long process to acquire the relevant knowledge and skills needed for their profession ( Ericsson, 2004). It is necessary for emergency physicians to gain the knowledge and skills to assess, stabilize, investigate, and manage critical and acute cases.

Emergency departments accessibility to dental and oral surgery services differs depending on the medical institution. Emergency physicians may have full-time consultation privileges and little need to carry any dental procedures within their department. However, very often they will be the only providers available to manage patients presented with dental pain or injury ( Ryan & Stone, 2010).

Aside from hospital resources available, every emergency physician is expected to be competent in managing basic emergency dental procedures ( Amsterdam, 2009). This paper provides the first guidelines that outline the stages of developing an educational workshop that will target emergency medicine residents and physicians for the purpose of improving their knowledge and skills in managing several dental emergencies. It describes the theoretical steps that are usually completed before conducting the workshop. These steps include describing the context in which the workshop will take place, assessing residents’ and physicians’ needs, developing the design and the delivery strategy, and evaluating the learners and workshop ( Figure 1).

**Figure:**
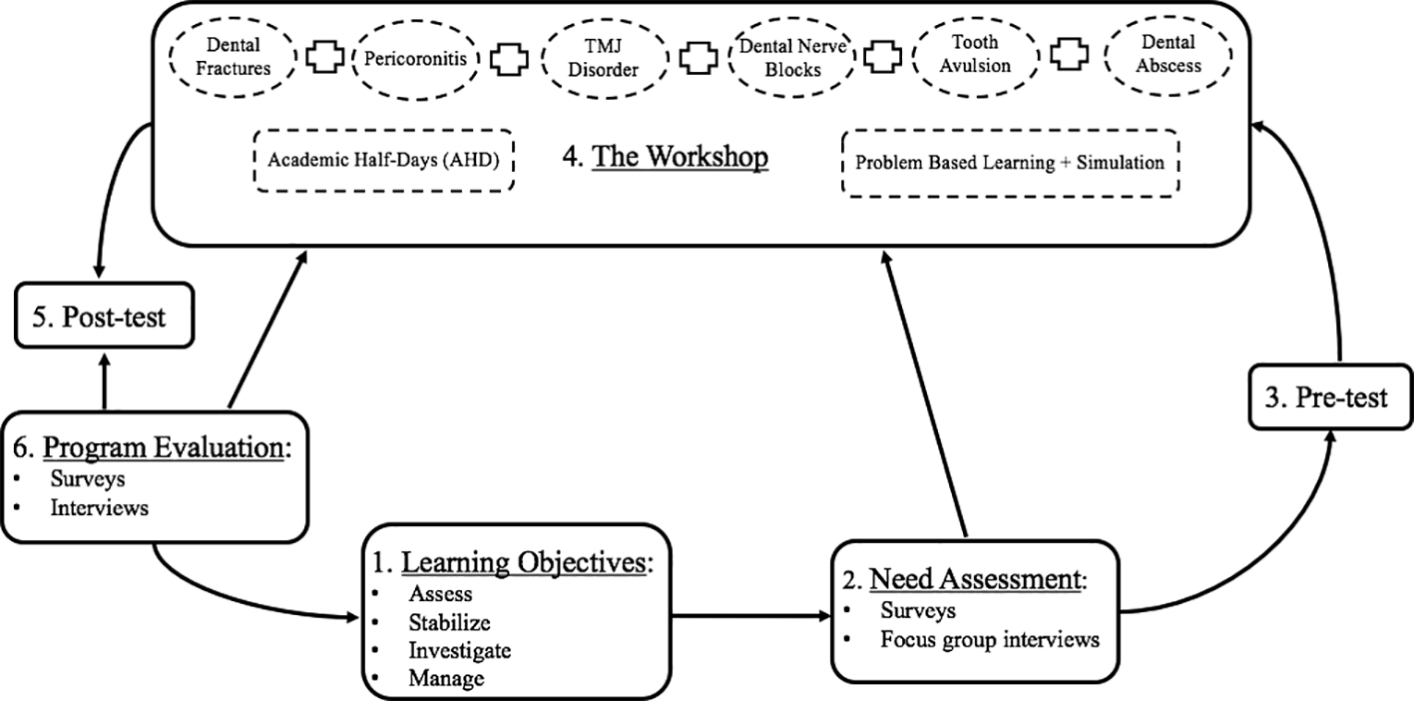


## Context

The Saudi Program of Emergency Medicine (SPEM) under the Saudi Commission for Health Specialties envisions becoming the strongest and most active academic department of emergency medicine in the Middle East. It offers a four-year residency program. Residents are offered a four-year rotating curriculum of core topics in emergency medicine during their course of study that lasts from years PGY1 to PGY4. Academic half day (AHD) rounds are scheduled weekly. Occasionally, special guests are invited to join these rounds, and skill developing workshops are also sometimes conducted. After the fourth year of the program, residents must pass the Saudi Board of Emergency Medicine Exam in order to graduate and be certified as emergency physicians. (www.scfhs.org.sa)

## Conducting need assessment

Dental emergency cases are rarely encountered in emergency departments because patients usually prefer to wait to see their dentists. However, some patients cannot tolerate the pain or may require emergency treatment due to accidents and major face trauma. Therefore, EM physicians require sufficient knowledge and skills to deal with such cases when they do arise even if they are infrequent ( Amsterdam, 2009)

The conduction of needs assessment is consistently advisable in order to develop relevant curriculum ( Aherne et al, 2001). For this proposed workshop, the use of surveys and a focus group interview is suggested. The questionnaire introduced in this paper is composed of five 4-point Likert questions and one open-ended question asking residents and physicians to describe one dental emergency case they have faced, how they felt about dealing with it, and their approach in managing the case ( Appendix A). Because of our target group’s busy schedule and because email is used as the official method of communication, the questionnaire will be emailed. Residents ranging from those in their first year of their EM residency program to those who are in their final year will be expected to participate as will EM physicians and consultants. A focus group open interview with chief residents and the program director will provide more insight regarding the types of dental emergencies usually encountered in the emergency department. The findings are expected to help demonstrate the need and obtain information that could be helpful in drafting the development of the workshop. There is no doubt that such a workshop will increase the confidence of emergency medicine residents and physicians when treating dental emergency cases.

## Designing and developing the workshop

Data collection and analysis from both questionnaire and focus group interviews will help in developing a preliminary list of relevant categories and themes. Designing and developing the workshop will involve deciding what content to include, writing the content, and gathering the learning material. The content of the teaching sessions should be relevant, interesting, and practical. Six themes are expected to emerge: 1.) dental abscess; 2.) pericoronitis; 3.) TMJ disorders; 4.) dental nerve blocks; 5.) dental fractures; and 6.) tooth avulsions ( Ryan & Stone, 2010).

Emergency medicine is a profession that deals with problems, diagnostic dilemmas, and variability in treatment plans and algorithms. Managing dental complaints is not a well-defined process. Therefore, teaching strategies that involve analysis and problem solving skills such as problem-based learning and simulation are essential components for this workshop.

Since problem-based learning was first introduced at McMaster University in the mid-1960’s, it has been extensively used in education. Problem-based learning (PBL) is the learning that happens during the process of understanding and solving a problem (Barrows & Tamblyn 1980). It is a pedagogic strategy in which facilitators present scenarios that allow learners to gain knowledge in a self-directed form ( Gidman & Mannix 2007). PBL creates an enjoyable learning environment, which promotes problem-solving skills in conjunction with communication and verbal skills. In addition, it gives learners the opportunity to discuss the moral and ethical dimensions of given scenarios ( Federnman 1999).

In addition, simulation is a teaching strategy that has been a favored teaching method in healthcare education for a while. It is used to reproduce elements of a real-life situations integrated in such a way in order to achieve specific goals. Simulation provides a risk-free environment that creates a safe hands-on setting. It promotes not only critical thinking and decision-making skills but also the clinical expertise needed to manage simple and complex clinical situations ( Decker et al 2008). Simulation comes in three levels of fidelity: 1.) low-; 2.) moderate-; and 3.) high-fidelity simulators. For this workshop, the use of low- and moderate-fidelity simulators is suitable.

Problem-based learning combined with simulation is expected to provide residents and physicians with critical thinking abilities in addition to the knowledge and the skills needed to effectively manage their cases.

## Workshop evaluation

Evaluation is composed of several organized steps taken to both gather and convey information about a delivered program ( Hawthorn & McDavid, 2006). For this proposed workshop, an evaluation should be conducted to measure the benefits of the program and to evaluate the workshop as a whole (context, design, and delivery). The use of multiple-choice questionnaires is common in assessments. It can be administrated in a relatively short-period of time and also covers several contents areas. As such, the administration of this evaluation format is straightforward and standardized ( Epstein, 2007). The questionnaire should ask learners to choose the best answer from a list of possible answers. Pre- and post- tests should be conducted before and after the workshop. The significance of the differences between each resident’s scores before and after the workshop will give insights into the program efficiency and effectiveness. Questions should address all six themes of the program and focus on assessing, stabilizing, investigating, and managing dental emergencies.

A modified version of the Learner Experience Feedback Form ( Appendix B) that was built to align with W(e)Learn can be used to evaluate the workshop. It is a framework developed to guide the design, delivery, development, and evaluation of interprofessional courses and programs (MacDonald, Stodel, Thompson, & Casimiro, 2009).

## Summary

Throughout this paper, we have explained the steps needed to conduct a dental emergency educational workshop, including a discussion of a need assessment, how to design and develop the workshop, and evaluation of learners and the workshop. These guidelines are general and can be used for planning any educational workshop.

## Recommendation

• Conducting a questionnaire to assess if there is an actual need for this proposed workshop
• Developing and conducting the workshop
• Continuous evaluation and improvement of the workshop

## Notes On Contributors

Nozha M. Sawan

Bacholar of Dental Surgery from King Saud University- Riyadh, Saudi Arabia (BDS)

Masters in Health Profession Education from University of Ottawa- Ottawa, Canada (MEd)

Masters in Dental Science from Indiana University- Indianapolis, USA (MSD)

Diplomate of the American Board of Orthodontics

Works at Princess Nourah Bint Abdulrahman University- College of Dentsitry, Department of Preventive Science, Division of Orthodontics

## Appendices

### Appendix A

You are about to participate in a training need survey. Please choose the answer that best reflects your level of satisfaction

#### Are you a:

____ Consultant ____ Staff physician ____ Senior resident ____ Junior resident

#### Are you obtaining or have obtained your training:

____ Locally (in Saudi Arabia) ____ Abroad (Europe) ____Abroad (North American)

____ Other, please specify______________

#### Satisfaction with your level of knowledge about dental emergencies:

• Very satisfied
• Satisfied
• Slightly dissatisfied
• Very dissatisfied

#### Satisfaction with your skills in assessing any case with a dental emergency:

• Very satisfied
• Satisfied
• Slightly dissatisfied
• Very dissatisfied

#### Satisfaction with your skills in stabilizing any case with a dental emergency:

• Very satisfied
• Satisfied
• Slightly dissatisfied
• Very dissatisfied

#### Satisfaction with your skills in managing any case with a dental emergency:

• Very satisfied
• Satisfied
• Slightly dissatisfied
• Very dissatisfied

#### Satisfaction with the amount of information about dental emergencies in your current curriculum:

• Very satisfied
• Satisfied
• Slightly dissatisfied
• Very dissatisfied

#### Describe one dental emergency that you have faced. How did you manage the emergency? Did you feel comfortable dealing with the case?

Thank you for your participation!

### Appendix B Learning Experience Feedback Form

The information you provide here will be kept completely confidential. Results will be reported in a group format and no individually identifying information will be included. Only the Evaluation Team will have access to this information. Please rate how much you agree or disagree with the following statements by checking the answer that best reflects your experience with the Dental Emergencies workshop:

**Table:**

	Strongly disagree	Disagree	Neutral	Agree	Strongly agree
**Content**					
The objectives of the workshop were made clear					
The workshop was well organized					
The workshop was of appropriate depth and breadth					
The workshop included information that I will be able to use to deal with dental emergencies in the workplace					
The workshop included information that I will be able to use to deal with dental emergencies					
The workshop addressed learning situations similar to those I face at work					
The workshop covered current best practices in dental emergencies					
**Delivery**					
I received useful feedback from the facilitators					
The workshop was presented in an interesting manner					
The workshop was presented in an interactive manner					
There was sufficient variety in the way the content was presented					
**Service**					
Facilitators responded quickly to suggestions made by learners					
Facilitators responded quickly to complaints made by learners					
Facilitators were knowledgeable about dental emergencies					
**Structure**					
The workshop kept my interest					
The workshop built my confidence in understanding how to deal with dental emergencies					
The workshop contained realistic and relevant cases					
There was a positive learning environment					
**Outcomes**					
I enjoyed the experience					
As a result of attending this workshop I can correctly assess patients with dental complaints					
As a result of attending this workshop I can stabilize patients with dental traumas and pain					
As a result of attending this workshop I can properly manage patients with dental emergencies					
