# Supplementary material for: Guidelines for the development of a dental emergencies educational workshop
Source: MedEdPublish (2016). 2017 Jun 14;6:101. [Version 1] doi: 10.15694/mep.2017.000101 (PMC10885263; doi:10.15694/mep.2017.000101)
Supplement: Peer review history

# Reviewer report: Reviewer response for version 1

**DOI:** 10.21956/mep.19260.r27982

**Published:** 2019-04-03

**Authors:** Ken Masters

**Affiliations:** 1 Sultan Qaboos University

This review has been migrated. The reviewer awarded 3 stars out of 5 This is an interesting paper, but I feel that the author has not completed all the steps.The model outlined in the Introduction, does, indeed, give a comprehensive overview of steps to be followed in conducting a useful workshop. When the author fleshes it out, however, too much information is missing:• There is no discussion of the Learning Objectives creation• Structurally, the pre-test should be described earlier, and its relationship to the Objectives and Needs Assessment clearly defined.• The relationship between the post and pre test should be discussed – for example, are they to be the same or different, and the advantages and disadvantages of each. In addition, while “significant differences” is a useful start, the authors might wish to choose a model for assessment of differences, or at least give more criteria (e.g. on how many items would a significant difference be considered a success).So, I think that the paper is of interest to many, but the author has a little more work to do on fleshing out the activities described in the model.

Reviewer Expertise:

NA

No decision status is available

# Reviewer report: Reviewer response for version 1

**DOI:** 10.21956/mep.19260.r27983

**Published:** 2019-03-07

**Authors:** Trevor Gibbs

**Affiliations:** 1 AMEE

This review has been migrated. The reviewer awarded 4 stars out of 5 An interesting paper which is written very clearly and is based upon need. I feel that it will prove useful to all those involved in dental emergencies.My only other suggestion regarding the paper would be to arrange a more longitudinal evaluation that looks at how these guidelines affected the real life work experiences.

Reviewer Expertise:

NA

No decision status is available

# Reviewer report: Reviewer response for version 1

**DOI:** 10.21956/mep.19260.r27980

**Published:** 2017-07-26

**Authors:** Zain Gulzar

**Affiliations:** 1 Institute of Advanced Dental Sciences and Research

This review has been migrated. The reviewer awarded 4 stars out of 5 This paper clearly and concisely describes the guidelines for designing an educational workshop on the topic of dental emergencies handling, by emergency medicine department’s residents and physicians. It’s essential for emergency medicine department residents and physicians to have basic knowledge and skills to assess, stabilize, investigate, and manage critical and acute cases. Teaching strategies like problem bases learning combined with stimulation are better choices to acquire competencies to manage dental emergencies, facing real life situations in risk free environment.This paper is informative and these guidelines are helpful for those who are designing and conducting the continuing professional development workshops. However, validity of these guidelines needs to be searched upon to make it implementable in various situations.Learner and workshop evaluation questionnaire is generic that can use for evaluation of any workshop after making some modifications and changes in it.

Reviewer Expertise:

NA

No decision status is available

# Reviewer report: Reviewer response for version 1

**DOI:** 10.21956/mep.19260.r27981

**Published:** 2017-07-19

**Authors:** John Dent

**Affiliations:** 1 AMEE

This review has been migrated. The reviewer awarded 3 stars out of 5 This paper offers guidelines for the development of an educational workshop in dental emergencies targeting the needs of residents and physicians in Emergency Medicine.The guidelines are enumerated and described in clearly presented sections making the ideas easy to identify and the method of implementation easy to follow. A useful flow-diagram is supplied together with questionnaires for the use of participants before and after the workshop. The paper gives clear guidelines to anyone wishing to implement a workshop in this field and the generic nature of the instructions makes them relevant for anyone considering providing an educational workshop.Unfortunately, I could not trace one of the references supplied either because of misspelling or because of insufficient citation details.

Reviewer Expertise:

NA

No decision status is available
